# Prevalence of double burden of malnutrition among children cared for in primary health care units: a descriptive study, Icapuí, Ceará, 2023

**DOI:** 10.1590/S2237-96222026v35e20250186.en

**Published:** 2026-04-03

**Authors:** José Tércio Pereira de Carvalho, Géssica de Souza Martins, Carla Soraya Costa Maia, Maria Dinara De Araújo Nogueira

**Affiliations:** 1 Escola de Saúde Pública do Ceará, Fortaleza, CE, Brazil Escola de Saúde Pública do Ceará Fortaleza CE Brazil; 2 Universidade Estadual do Ceará, Fortaleza, CE, Brazil Universidade Estadual do Ceará Fortaleza CE Brazil

**Keywords:** Pediatric Obesity, Malnutrition, Food and Nutritional Surveillance, Public Health, Observational Study., Obesidad Infantil, Malnutrición, Vigilancia Alimentaria y Nutricional, Salud Pública, Estudio Observacional

## Abstract

**Objective:**

To identify the nutritional status and the prevalence of the double burden of malnutrition among children cared for in primary health care units in Icapuí, Ceará.

**Methods:**

A descriptive study was conducted with anthropometric data analysis, using data from the Brazilian Food and Nutrition Surveillance System of 2023. The variables used were weight, height, and body mass index (BMI). Nutritional status was classified according to the World Health Organization growth curves, using WHO Anthro-Plus 2007 software. Double burden was defined as the simultaneous occurrence of two alterations: stunting and High BMI-for-age. Numerical variables are presented as means and standard deviations, while categorical variables are presented as frequencies and percentages.

**Results:**

Of the 1,704 children aged zero to nine years evaluated, 23.9% presented excess weight (overweight, obesity, or severe obesity) and 10.2% were short or very short in stature. The double burden of malnutrition was observed in 4.7% of the children, being more prevalent in those under 5 years old (6.5%) than in those aged 5-9 years (3.0%). Boys had a higher prevalence of stunting and double burden compared to girls.

**Conclusion:**

The double burden of malnutrition is an epidemiological reality in Icapuí, with distinct patterns: children under 5 years old are more vulnerable to stunting, whereas those aged 5-9 years are more affected by excess weight; boys are at greater risk of stunting, while girls are at greater risk of overweight.

## Resumen

**Objetivo:**

Identificar el estado nutricional y la prevalencia de la doble carga de malnutrición en niñas y niños atendidos en unidades de atención primaria de salud en Icapuí, Ceará.

**Métodos:**

Se realizó un estudio descriptivo con análisis de datos antropométricos, utilizando información del Sistema Brasileño de Vigilancia Alimentaria y Nutricional correspondiente al año 2023. Las variables utilizadas fueron peso, talla e índice de masa corporal (IMC). El estado nutricional se clasificó según las curvas de crecimiento de la Organización Mundial de la Salud, mediante el software WHO Anthro-Plus 2007. La doble carga se definió como la ocurrencia simultánea de dos alteraciones: baja talla para la edad y alto IMC para la edad. Las variables numéricas se presentan como medias y desviaciones estándar, y las variables categóricas como frecuencias y porcentajes.

**Resultados:**

De las 1.704 niñas y niños de cero a nueve años evaluados, el 23,9% presentó exceso de peso (sobrepeso, obesidad u obesidad grave) y el 10,2% mostró talla baja o muy baja. La doble carga de malnutrición se observó en el 4,7% de las niñas y los niños, siendo más prevalente en los menores de 5 años (6,5%) que en los de 5 a 9 años (3,0%). Los niños presentaron una mayor prevalencia de baja talla y de doble carga en comparación con las niñas.

**Conclusión:**

La doble carga de malnutrición constituye una realidad epidemiológica en Icapuí, con patrones distintos: las niñas y los niños menores de 5 años son más vulnerables al retraso en el crecimiento, mientras que aquellos de 5 a 9 años se ven más afectados por el exceso de peso; los niños presentan mayor riesgo de baja talla y las niñas, de sobrepeso.

**Ethical aspects**

This research respected ethical principles, having obtained the following approval data:

Research ethics committee: Escola de Saúde Pública do Ceará

Opinion number: 6992721

Approval date: 8/8/2024

Certificate of submission for ethical appraisal: 81036624.2.0000.5037

Informed consent record: Not applicable

## Introduction 

The double burden of malnutrition, characterized by the coexistence of overweight and stunting, as well as micronutrient deficiencies and other conditions related to inadequate nutrition, is a public health problem of global significance and a cause for concern for the World Health Organization ^1^. Although initially described in adults, this double burden considerably affects children, with health consequences that may extend throughout life. In low- and middle-income countries, the magnitude of this condition remains poorly defined, especially in light of recent changes in population eating habits ^2^.

It is estimated that, globally, 41 million children under five years old are overweight or obese. At the same time, growth delays affected 149 million children in the same age group in 2020, and malnutrition was associated with about 45% of deaths among children under five years old ^3^. In Brazil, recent data indicate that 9.5% of children are stunted, while 15.8% are overweight ^4^.

Recent studies show that although the prevalence of malnutrition has decreased, overweight has increased among children under five years old. Nevertheless, research focused on the Northeast region, which is home to a large portion of Brazil's low-income population and faces high rates of food insecurity, is scarce. This issue is concerning, as children in less developed regions of Brazil are more vulnerable to the double burden of malnutrition ^5^.

This situation is also observed in smaller cities in terms of population and economy, such as Icapuí, in Ceará, based on data from the Brazilian Food and Nutrition Surveillance System (*Sistema de Vigilância Alimentar e Nutricional* - SISVAN), an important monitoring tool. According to SISVAN, about half of children up to nine years old are overweight. However, these data do not show the coexistence of overweight and stunting, thus making it difficult to identify children who have both parameters altered simultaneously ^6^. Icapuí has 100% population coverage by primary health care teams, especially those focused on family and community health ^7^. This coverage enables not only in-depth studies but also the implementation of integrated care and prevention strategies.

In this context, the objective of this study was to identify the nutritional status and the prevalence of the double burden of malnutrition among children cared for in primary health care units in Icapuí, Ceará. Further investigation of this issue is essential to support more effective strategies to address nutritional challenges and to support public policies that promote the health and well-being of children ^4^.

## Methods 

Study Design

This is a descriptive study using population data from SISVAN reports for the year 2023 in Icapuí, a coastal municipality in Ceará, a state in the Northeast region of Brazil.

Setting

Icapuí was identified as the easternmost municipality in the state of Ceará, with an estimated population of 21,400 inhabitants, according to the Brazilian Institute of Geography and Statistics (*Instituto Brasileiro de Geografia e Estatística* - IBGE) in the 2022 census ^8^. Of this total, approximately 1,940 were children between the ages of zero and nine. Most of the population was concentrated in the coastal area, and the local economy was based on agriculture and fishing, including the capture of lobster, shrimp, and shellfish.

Participants

The study population consisted of children aged 0 to 9 years, of both sexes, with anthropometric records entered in the Brazilian Food and Nutrition Surveillance System (*Sistema de Vigilância Alimentar e Nutricional* - 

SISVAN Web ) between January and December 2023 ^6^. The data was accessed through a restricted manager profile on the official platform of the Brazilian Ministry of Health (available from: https://sisaps.saude.gov.br/sisvan/). A professional duly accredited by the municipality performed data extraction in 2024.

For sample composition, filters from the Anthropometric Reports module referring to the period from January to December 2023 and the age group from 0 to 9 years were applied. The consolidated report, containing the number of individuals assessed and the anthropometric information, was exported for further analysis. All children with incomplete or inconsistent anthropometric records were excluded from the final sample.

Variables

The following variables were analyzed: anthropometric data on weight (kg), height (cm), and body mass index (BMI, calculated as weight/height² in kg/m²). Nutritional status was classified according to the World Health Organization (WHO) parameters, using the indicators weight-for-age, weight-for-height (the latter applicable only to children under five years old), height-for-age, and BMI-for-age, all assessed by Z-scores. For the analysis, children were initially stratified into five age groups (0-6 months; 6 months to <2 years; 2 to <5 years; 5 to <7 years; 7 to <10 years), and later regrouped into under five years old and 5-9 years old. The primary outcome was the presence of the double burden of malnutrition, defined as the coexistence of excess weight (overweight or obesity, according to the BMI-for-age classification) and stunting (height-for-age Z-score <-2).

Data sources and measurement

The database included information on weight, height, and body mass index (BMI), calculated as weight divided by height squared. Using the WHO Anthro-Plus 2007 software, classifications were obtained for the four nutritional status parameters recommended by the WHO ^9^: weight-for-age, weight-for-height (only for children under five years of age), height-for-age, and BMI-for-age, all based on the Z-score. The age groups analyzed were as follows: 0 to 6 months; 6 months to <2 years; 2 to <5 years; 5 to <7 years; and 7 to <10 years. For analysis purposes, children were grouped into those under five years old and those aged 5-9 years. After determining nutritional status, the presence of the double burden of malnutrition as the outcome was defined as the coexistence of excess weight (overweight or obesity) and stunting, identified based on BMI-for-age and height-for-age indicators.

Bias

As this was a study based on secondary data, potential biases inherent to this approach were considered, such as errors in data collection, entry, or recording. To minimize these biases, several strategies were adopted, including systematic consistency and completeness analyses, as well as identification and exclusion of records with missing, implausible, or inconsistent values. These procedures are consistent with literature recommendations for minimizing bias in studies using secondary data.

Study size

The study included all children aged zero to nine years (n=1,707) with anthropometric data recorded in SISVAN in Icapuí during the year 2023. After excluding incomplete or inconsistent records, the final sample comprised 1,704 children.

Statistical methods

The data collected were stored in Microsoft Excel 2019 and analyzed using the Statistical Package for the Social Sciences (SPSS), version 22.0. Numerical variables are presented as means and standard deviations, while categorical variables are presented as frequencies and percentages. Nutritional status and the prevalence of the double burden of malnutrition were stratified by age group and sex and presented in tables and graphs generated using GraphPad Prism 8.0 software.

## Results 

A total of 1,704 children were assessed, of whom 51.2% were female. The mean age was 59.23 months (SD 33.67), with a higher proportion of children aged 5-9 years (52.5%). Mean values for weight, height, and BMI were 20.62 kg (SD 9.94), 105.70 cm (SD 22.85), and 17.66 kg/m² (SD 3.99), respectively.

Analysis of weight-for-height showed that most children had adequate weight, although a considerable proportion presented risk of overweight (22.8%) or excess weight (overweight and obesity; 23.9%), totaling 46.7%. Regarding height-for-age, most children had adequate height. In the weight-for-age assessment, although most children had adequate weight, 19.4% presented high weight-for-age. The BMI-for-age evaluation indicated that 15.5% of the children were overweight, 13.6% were obese, and 4.8% were severely obese (Table 1).

Table 1 also provides a detailed analysis of nutritional status according to age group. Most children presented adequate height-for-age in both age groups. Regarding weight-for-age, high weight was more prevalent among children aged 5-9 years (22.9%). Regarding BMI-for-age, it was observed that, although the risk of overweight was more frequent in younger children (22.2%), excess weight was more prevalent among children aged 5-9 years, of whom 17.7% were overweight, 15% were obese, and 9.1% were severely obese.

Figure 1 illustrates the weight-for-height classification of children under five years of age, with overweight risk being more frequent among girls (27.4%). Overweight is also slightly higher among girls (14.0% versus 11.0% among boys). Obesity, in turn, was more prevalent among males (12.9%).

Concerning height-for-age by sex, most children had adequate height in both sexes and age groups.

Nonetheless, low and very low height were more frequent among boys in both age groups. In the weight-for-age classification, high weight was more prevalent among girls, especially those under five years old (17.8%). Among children aged 5-9 years, prevalence rates were similar between sexes (23.4% in girls versus 22.5% in boys) (Figure 2).

In Figure 3, which assesses BMI-for-age, the overweight risk, overweight, and obesity were more prevalent in girls under five years of age, with emphasis on the overweight risk (26.6%). In the case of children aged 5-9, overweight risk, overweight, and obesity were higher among boys, with particular emphasis on overweight (18.7%) and severe obesity (10.5%).

Regarding the double burden of malnutrition, the total prevalence found in the sample was 4.7%. When stratified by age group, a higher frequency of double burden of malnutrition was observed among children under five years old (6.5%). Among this group, the double burden of malnutrition was more prevalent among boys in both age groups. With regard to the parameters used in the classification of the double burden of malnutrition, excess weight was slightly more frequent among children aged 5-9 years, with similar prevalence between sexes. Stunting was more prevalent among children under five years old, particularly boys (17.5%) (Figure 4).

**Table 1:** Classification of the nutritional status of children treated at primary health care centers according to age group. Icapuí, Ceará, 2023 (n=1704)

Variables	Total	< 5-year-old children	5-9-year-old children	n (%)	n (%)	n (%)
Weight-for-height^a^			
Wasting	27 (3.3)	27 (3.3)	-
Thinness	29 (3.6)	29 (3.6)	-
Eutrophy	374 (46.2)	374 (46.2)	-
Overweight risk	184 (22.8)	184 (22.8)	-
Overweight	102 (12.6)	102 (12.6)	-
Obesity	91 (11.3)	91 (11.3)	-
Height-for-age			
Very low	94 (5.5)	49 (6)	45 (5)
Low	80 (4.7)	50 (6.2)	30 (3.4)
Appropriate	1530 (89.8)	711 (87.8)	819 (91.6)
Weight-for-age			
Very low	40 (2.3)	11 (1.4)	29 (3.2)
Low	35 (2.1)	20 (2.5)	15 (1.7)
Appropriate	1289 (76.2)	653 (80.6)	645 (72.1)
High	331 (19.4)	126 (15.6)	205 (22.9)
BMI-for-age^b^			
Wasting	48 (2.8)	33 (4.1)	15 (1.7)
Thinness	55 (3.2)	24 (3)	31 (3.5)
Eutrophy	814 (47.8)	370 (45.7)	444 (49.7)
Overweight risk	211 (12.4)	180 (22.2)	31 (3.5)
Overweight	264 (15.5)	106 (13.1)	158 (17.7)
Obesity	231 (13.6)	97 (12)	134 (15)
Severe obesity^c^	81 (4.8)	-	81 (9.1)

a Parameter available only for children under 5 years of age (n=807); ^b^Body mass index-for-age; ^c^Classification available only for children aged 5 years and older.

**Figure 1:**
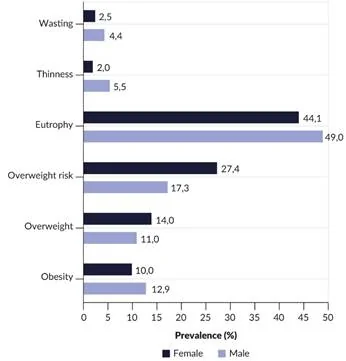
Classification of weight-for-height of children under five years of age (parameter available only for this age group) treated at primary healthcare units, according to sex. Icapuí, Ceará, 2023 (n=807)

**Figure 2:**
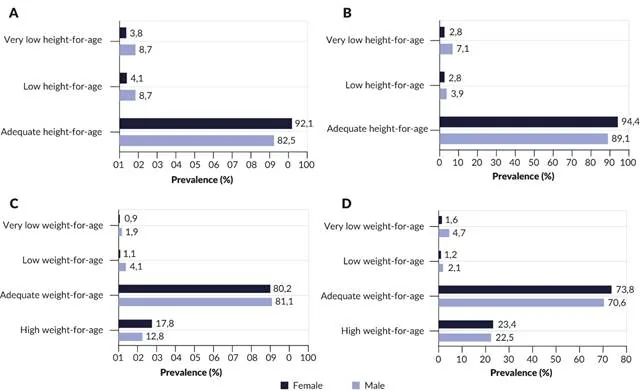
Height-for-age classification of children under five (A) and 5-9 years (B); weight-for-age classification of children under five (C) and 5-9 years (D) seen at primary health care units, according to sex. Icapuí, Ceará, 2023 (n=1704

**Figure:**
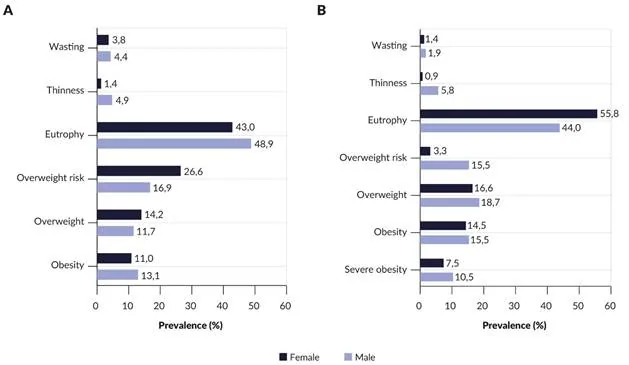
3. Classification of body mass index-for-age (BMI-for-age) of children under five (A) and 5-9 years (B) treated at primary health care units, according to sex. Icapuí, Ceará, 2023 (n=1704)

**Figure:**
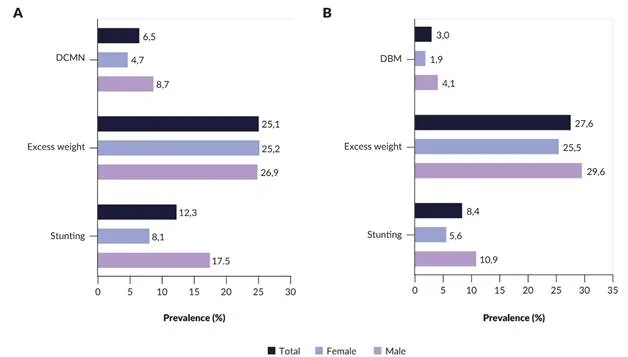
4. Prevalence of Double Burden of Malnutrition (DBM) and DBM classification criteria for children under five (A) and 5-9 years old (B) treated at primary health care units, according to sex. Icapuí, Ceará, 2023 (n=1704)

## Discussion 

This study revealed a significant prevalence of the double burden of malnutrition among children, highlighting the coexistence of opposite nutritional conditions, such as overweight and stunting. This survey represents a pioneering study in Ceará, with a relevant sample from the state's countryside, standing out for exploring the complex coexistence of childhood malnutrition conditions and their relationship with contextual and current trends ^10^.

The limitations inherent in studies using secondary data are well known. These relate to inconsistencies and incompleteness in some of the data collected, as well as the lack of direct integration between information on children and mothers (including their nutritional and socioeconomic status), which hindered associations between the determinants of the double burden of malnutrition ^11^. Even so, it is important to consider that the methodological choice of using exclusively BMI-for-age as a weight indicator for all age groups, although aligned with SISVAN recommendations and consistent with similar studies, may have generated specific differences in relation to other indicators (such as weight-for-age for children under two years of age). The decision was made to ensure comparability between age groups and to allow for the simultaneous assessment of short stature and overweight in the entire sample. Furthermore, since SISVAN only encompasses data from children who are attended in the public health system, it may affect the generalization of results, even though its national coverage has been expanding ^12^. Regarding data quality, the standardized protocols of the 2011 Technical Standard ^13^ ensure greater reliability of anthropometric measurements, even with possible variations in data collection.

In light of these findings although specific studies on the global prevalence of the double burden of malnutrition are limited, regional data provide important insights. For instance, in Africa, a study conducted in two Nigerian states with 1,200 children and adolescents found a prevalence of stunting of 34.9% and overweight/obesity of 11.4%. Thus, 4.0% of individuals presented both conditions simultaneously, varying according to demographic, socioeconomic, and family characteristics ^14^. In South America, recent surveys of children and adults found that the prevalence of double burden malnutrition in households in Bolivia was 9.3%, while in Peru it was 6.7%. In Colombia, the prevalence was 3.2%, while in Brazil it was 2.2% ^15^.

In Brazil, maternal conditions, such as nutritional status and educational level, influence the nutritional status of children, contributing to the double burden of malnutrition, as evidenced in a study involving children under five years of age in different regions of the country, including urban and rural areas ^16^.

In northeastern Brazil, studies analyzing data from 50,778 children and adolescents (0-14 years) showed mean prevalence rates of undernutrition, overweight, and obesity of 1.9%, 12.5%, and 7.4%, respectively. These studies also indicated socioeconomic factors such as housing and income as being associated with nutritional status ^17^.

In a cross-sectional study conducted in two municipalities in Paraíba, of the 413 children who participated in the study, 9.4% were diagnosed with stunting and 16.5% with overweight/obesity. Therefore, stunting in children can be associated with birth weight, gestational age, maternal nutritional status, and family socioeconomic status. As for overweight, it was found to be related to the child's age, maternal BMI, benefits from the *Bolsa Família* program, and social support ^5^^,^^18^.

The present study found that most children in Icapuí had adequate weight, although a considerable proportion presented overweight or obesity, especially in older age groups. Furthermore, stunting an indicator of chronic undernutrition was more frequent among children under five years, emphasizing the need for interventions to improve health conditions and ensure adequate growth from early childhood ^19^^,^^20^. In terms of age differences, the prevalence of the double burden of malnutrition was higher among children under five years than among those aged 5-9 years. The influence of sex was also evident, with males showing a higher prevalence of double burden, overweight, and stunting in both age groups. Such a disparity may be related to biological or cultural differences that affect nutritional health between boys and girls; further studies are needed to explore dietary practices, access to food, and general care ^21^^-^^23^.

Among older children, overweight was more prevalent, suggesting that disproportionate weight gain tends to occur at later ages. In under-five-year-olds, there was a higher prevalence of thinness and stunting, which can be attributed to socioeconomic factors and limited access to health services ^10^.

Both undernutrition and overweight components of the double burden are often associated with food insecurity, which leads families to consume foods with low nutritional value and high caloric density, such as ultra-processed products. This contributes to nutrient deficiencies and impaired growth, as well as excessive weight gain. It is a situation that reflects the ongoing nutritional and epidemiological transition, marked by changes in dietary patterns and sedentary lifestyles ^24^^-^^27^.

With regard to impaired linear growth, results indicated greater vulnerability among boys across both age groups. This situation may be explained by biological factors, such as higher susceptibility to adverse conditions in early life, or environmental ones, such as unequal access to nutritious foods and health services ^22^.

Among girls, the high rates of overweight may be associated with biological predisposition to fat accumulation, as well as cultural and social aspects such as less encouragement for physical activity. Emotional eating behaviors also contribute, as does the lack of inclusive preventive policies that take gender differences into account ^25^.

The persistence of growth deficits among a considerable proportion of children especially those in contexts of social vulnerability highlights the importance of public policies aimed at food and nutrition security, with special focus on early childhood and on regions with limited access to nutritious food ^28^. Given this situation, it is necessary to take action aimed at promoting both a balanced diet and regular physical activity. This is especially true in schools, which play an important role in promoting healthy eating habits and controlling the consumption of hypercaloric foods. These socioeconomic and environmental factors reinforce the need for an integrated approach to prevent both overweight and undernutrition ^21^^,^^13^.

This study stands out for its comprehensiveness, covering about 88% of the municipality's child population a significant scope compared to the total number of children living in Icapuí ^7^. Moreover, it represents an important milestone in investigating the double burden of malnutrition in northeastern Brazil, particularly in the countryside of Ceará, filling a significant gap in regional literature.

The findings of this study highlight the need for integrated and continuous interventions that address both the prevention and treatment of nutritional problems, with a focus on vulnerable populations. Public policies should prioritize the promotion of food and nutritional security, encourage healthy eating habits from childhood, and expand the coverage of programs such as SISVAN, integrating strategies aimed at nutrition education, access to healthy foods, and encouraging physical activity. Schools, in particular, can serve as catalysts for change toward healthier behaviors among children ^16^^,^^17^.

In conclusion, the double burden of malnutrition is an epidemiological reality among children assisted by the public health system in Icapuí, Ceará, with distinct patterns: children under five years are more vulnerable to stunting, whereas school-age children (5-9 years) are more prone to overweight. Moreover, gender disparities were observed, with boys being at higher risk of stunting and girls being at higher risk of overweight. These findings provide evidence to support the design of more effective interventions, emphasizing the need for coordinated responses that address both traditional and emerging nutritional challenges among children in northeastern Brazil. The complexity of the double burden calls for innovative approaches that go beyond conventional solutions, integrating local knowledge with scientific evidence to develop strategies capable of reversing this situation.

## Data Availability

Data supporting the results of this study are available upon request from the corresponding author.
