# Supplementary material for: Prevalence of double burden of malnutrition among children cared for in primary health care units: a descriptive study, Icapuí, Ceará, 2023
Source: Epidemiol Serv Saude. 2026 Apr 3;35:e20250186. doi: 10.1590/S2237-96222026v35e20250186.en (PMC13686300; doi:10.1590/S2237-96222026v35e20250186.en)
Supplement: Translation

# Translation: Prevalência de dupla carga de má nutrição em crianças atendidas nas unidades básicas de saúde: estudo descritivo, Icapuí, Ceará, 2023

**Authors:** José Tércio Pereira de Carvalho, Géssica de Souza Martins, Carla Soraya Costa Maia, Maria Dinara De Araújo Nogueira

**Affiliations:** 1 Escola de Saúde Pública do Ceará, Fortaleza, CE, Brasil Escola de Saúde Pública do Ceará Fortaleza CE Brasil; 2 Universidade Estadual do Ceará, Fortaleza, CE, Brasil Universidade Estadual do Ceará Fortaleza CE Brasil

**Keywords:** Obesidade Infantil, Desnutrição, Vigilância Alimentar e Nutricional, Saúde Pública, Estudo Observacional.

## Abstract

**Objetivo:**

Identificar o estado nutricional e a prevalência da dupla carga de má nutrição em crianças atendidas nas unidades básicas de saúde de Icapuí, Ceará.

**Métodos:**

Foi realizado um estudo descritivo com análise de dados antropométricos, utilizando dados do Sistema de Vigilância Alimentar e Nutricional de 2023. As variáveis utilizadas foram peso, altura e índice de massa corporal (IMC). A partir do software WHO Anthro-Plus 2007, foi classificado o estado nutricional das crianças de acordo com as curvas da Organização Mundial da Saúde. Para determinação da dupla carga, foram consideradas duas alterações simultâneas: baixa estatura-para-idade e excesso de peso pelo IMC-para-idade. As variáveis numéricas são apresentadas em médias e em desvios-padrão, enquanto as variáveis categóricas são apresentadas em frequências e em percentuais.

**Resultados:**

Das 1.704 crianças de zero a nove anos avaliadas, 23,9% apresentaram excesso de peso (sobrepeso, obesidade ou obesidade grave) e 10,2% demonstraram baixa ou muito baixa estatura. A dupla carga de má nutrição foi observada em 4,7% das crianças, sendo mais prevalente nos menores de cinco anos (6,5%) do que nos de 5-9 anos (3,0%). Meninos apresentaram maior prevalência de baixa estatura e dupla carga em comparação às meninas.

**Conclusão:**

A dupla carga de má nutrição é uma realidade epidemiológica em Icapuí, com padrões distintos: menores de cinco anos são mais vulneráveis ao déficit estatural, e as de 5-9 anos, ao excesso de peso; meninos têm maior risco de baixa estatura, e meninas, de sobrepeso.

Ethical aspects

This research respected ethical principles, having obtained the following approval data:

Research ethics committee Escola de Saúde Pública do Ceará

Opinion number: 6,992,721

Approval date: 8/8/2024

Certificate of submission for ethical appraisal: 81036624.2.0000.5037

Informed consent record: Not applicable.

## Introdução 

A dupla carga da má nutrição, caracterizada pela coexistência de excesso de peso e de baixa estatura, além de deficiências de micronutrientes e outras condições relacionadas à alimentação inadequada, é um problema de saúde pública de relevância global e que gera preocupação na Organização Mundial de Saúde ^1^. Apesar de originalmente descrita em adultos, essa dupla carga afeta consideravelmente crianças, com consequências para a saúde que podem se estender ao longo de toda a vida. Em países de baixa e de média renda, a magnitude dessa condição permanece pouco definida, especialmente diante das mudanças recentes nos hábitos alimentares da população ^2^.

Estima-se que, globalmente, 41 milhões de crianças menores de cinco anos apresentam sobrepeso ou obesidade. Paralelamente, atrasos no crescimento afetaram 149 milhões de crianças do mesmo grupo etário em 2020, e a desnutrição foi associada a cerca de 45% das mortes de menores de cinco anos ^3^. No Brasil, dados recentes apontam que 9,5% das crianças apresentam baixa estatura, enquanto 15,8% estão com excesso de peso ^4^.

Estudos recentes indicam que, embora a prevalência de desnutrição tenha diminuído, o excesso de peso tem aumentado entre crianças menores de cinco anos. No entanto, há uma escassez de pesquisas focadas na região Nordeste, que concentra uma grande parcela da população brasileira de baixa renda e enfrenta altos índices de insegurança alimentar. Essa questão é preocupante, pois crianças de regiões menos desenvolvidas do Brasil apresentam maior vulnerabilidade à dupla carga de má nutrição ^5^.

Esse cenário também é observado em cidades de menor porte em termos de população e de economia, como Icapuí, no Ceará, através do Sistema de Vigilância Alimentar e Nutricional (SISVAN), uma importante ferramenta de monitoramento. Pelo SISVAN, cerca de metade das crianças até nove anos apresentam sobrepeso. No entanto, esses dados não mostram a coexistência de excesso de peso e de baixa estatura, tornando difícil a identificação de crianças que têm os dois parâmetros alterados simultaneamente ^6^. Icapuí conta com 100% de cobertura da população pelas equipes de Atenção Primária à Saúde, especialmente aquelas voltadas à saúde da família e da comunidade ^7^. Essa cobertura possibilita não apenas estudos aprofundados, mas também a implementação de estratégias integradas de cuidado e de prevenção.

Diante desse contexto, este estudo tem como objetivo identificar o estado nutricional e a prevalência da dupla carga de má nutrição em crianças atendidas nas Unidades Básicas de Saúde de uma Icapuí, Ceará. O aprofundamento nesse tema é essencial para apoiar estratégias mais eficazes no enfrentamento dos desafios nutricionais e para subsidiar políticas públicas que promovam a saúde e o bem-estar da população infantil ^4^.

## Métodos 

Delineamento do estudo

Trata-se de um estudo descritivo, utilizando dados populacionais dos relatórios do SISVAN referentes ao ano de 2023, em Icapuí, município litorâneo do Ceará, estado situado na região Nordeste do Brasil.

Contexto

Icapuí foi identificado como o município mais oriental do estado do Ceará, com uma população estimada em 21.400 habitantes, segundo o Instituto Brasileiro de Geografia e Estatística no censo de 2022 ^8^. Desse total, cerca de 1.940 eram crianças entre zero e nove anos de idade. A maior parte da população se concentrava na zona litorânea, e a economia local baseava-se na agricultura e na pesca, incluindo-se a captura de lagosta, de camarão e de mariscos.

Participantes

A população do estudo consistiu em crianças entre zero e nove anos, de ambos os sexos, com registros antropométricos cadastrados no Sistema de Vigilância Alimentar e Nutricional (SISVAN Web) no período de janeiro a dezembro de 2023 ^6^. Os dados foram acessados por meio de perfil restrito de gestor na plataforma oficial do Ministério da Saúde (disponível em: https://sisaps.saude.gov.br/sisvan/). Um profissional devidamente credenciado pelo município realizou a extração dos dados em 2024.

Para a composição da amostra, aplicaram-se os filtros do módulo Relatórios Antropométricos referentes ao período de janeiro a dezembro de 2023 e à faixa etária de zero a nove anos. O relatório consolidado, contendo a quantidade de indivíduos avaliados e as informações antropométricas, foi exportado para análise subsequente. Foram excluídas da amostra final todas as crianças com registros antropométricos incompletos ou inconsistentes.

Variáveis

Foram analisadas as seguintes variáveis: dados antropométricos de peso (kg), altura (cm) e índice de massa corporal (IMC, calculado como peso/altura² em kg/m²). O estado nutricional foi classificado segundo os parâmetros da Organização Mundial de Saúde (OMS), utilizando-se os indicadores peso-para-idade, peso-para-estatura (este último aplicável apenas para crianças menores de cinco anos), estatura-para-idade e IMC-para-idade, todos avaliados por meio do escore-Z. Para análise, as crianças foram inicialmente estratificadas em cinco faixas etárias (0-6 meses; 6 meses a <2 anos; 2 a <5 anos; 5 a <7 anos; 7 a <10 anos), sendo posteriormente reagrupadas em menores de cinco anos e de 5-9 anos. O desfecho principal foi a presença de dupla carga de má nutrição, definida como a coexistência de excesso de peso (sobrepeso ou obesidade, conforme classificação pelo IMC-para-idade) e baixa estatura (estatura-para-idade <-2 escore-Z).

Fontes de dados/mensuração

No banco de dados foram obtidas informações de peso, altura e índice de massa corporal (IMC), calculado a partir do peso dividido pela altura ao quadrado. A partir do software WHO Anthro-Plus 2007 foram obtidas as classificações dos quatro parâmetros de estado nutricional preconizados pela OMS ^9^, peso-para-idade, peso-para-estatura (apenas para menores de cinco anos), estatura-para-idade e IMC-para-idade, todos a partir do Escore-Z. As faixas etárias analisadas foram as seguintes: 0 a 6 meses; 6 meses a <2 anos; 2 a <5 anos; 5 a <7 anos; 7 a <10 anos. Para fins de análise, as crianças foram agrupadas em menores de cinco anos e de cinco a nove anos. Após definição do estado nutricional, a presença de dupla carga de má nutrição como desfecho foi definida pela coexistência de excesso de peso (sobrepeso ou obesidade) e de baixa estatura, identificados por meio do IMC-para-idade e da estatura-para-idade.

Viés

Por se tratar de um estudo com dados secundários, foram considerados os potenciais vieses inerentes a essa abordagem, como erros na coleta, na digitação ou no registro dos dados. Para minimizá-los, adotamos algumas estratégias, como uma análise sistemática de consistência e de completude, com a identificação e a exclusão de registros contendo valores ausentes, implausíveis ou inconsistentes. Essas medidas estão alinhadas com as recomendações da literatura para mitigação de vieses em estudos que utilizam dados secundários.

Tamanho do estudo

O estudo incluiu todas as crianças de zero a nove anos (n=1.707) com dados antropométricos registrados no SISVAN em Icapuí durante o ano de 2023. Após a exclusão de registros incompletos ou inconsistentes, a amostra final foi composta por 1.704 crianças.

Métodos estatísticos

Os dados coletados foram armazenados no Microsoft Excel 2019 e analisados por meio do software Statistical Package for Social Science versão 22.0. As variáveis numéricas são apresentadas em médias e em desvios-padrão, enquanto as variáveis categóricas são apresentadas em frequências e em percentuais. O estado nutricional e a prevalência de dupla carga de má nutrição foram estratificados por faixa etária e por sexo e apresentados em tabelas e gráficos que foram desenvolvidos pelo software GraphPad Prism 8.0.

## Resultados 

Foram avaliadas 1.704 crianças, das quais 51,2% eram do sexo feminino. A média de idade foi de 59,23 meses (DP 33,67), com uma maior proporção de crianças na faixa etária de 5-9 anos (52,5%). Os valores médios para peso, altura e IMC foram 20,62 kg (DP 9,94), 105,70 cm (DP 22,85) e 17,66 kg/m² (DP 3,99), respectivamente.

A análise do peso-para-estatura mostrou que a maioria das crianças apresentavam peso adequado, mas com uma parcela preocupante de risco para sobrepeso (22,8%) e de excesso de peso (sobrepeso e obesidade; 23,9%), somando 46,7%. Com relação a estatura-para-idade, a maioria das crianças apresentaram estatura adequada. Ao analisar o peso-para-idade, constatou-se que, apesar de a maioria estar com o peso adequado, 19,4% das crianças apresentaram peso elevado para a idade. A avaliação do IMC-para-idade evidenciou que 15,5% apresentavam sobrepeso, 13,6% obesidade e 4,8% obesidade grave (Tabela 1).

A Tabela 1 também apresenta uma análise detalhada do estado nutricional de acordo com a faixa etária. A maioria das crianças apresentou estatura adequada para a idade em ambas as faixas etárias. No que se diz respeito ao peso-para-idade, o peso elevado foi mais prevalente entre as crianças de 5-9 anos (22,9%). Em relação ao IMC-para-idade, observou-se que, apesar de o risco de sobrepeso apresentar maior frequência em crianças menores (22,2%), o excesso de peso foi mais prevalente entre as crianças de 5-9 anos, em que 17,7% apresentaram sobrepeso, 15% obesidade e 9,1% obesidade grave.

A Figura 1 trata da classificação do peso-para-estatura das crianças menores de cinco anos, sendo o risco de sobrepeso mais frequente entre as meninas (27,4%), e o sobrepeso levemente maior também nas meninas (14,0% versus 11,0% nos meninos). A obesidade, por sua vez, foi mais prevalente no sexo masculino (12,9%).

Quanto à análise da estatura-para-idade estratificada por sexo, observou-se que a maioria das crianças apresentaram estatura adequada em ambos os sexos e ambas as faixas etárias.

Entretanto, destacaram-se a baixa e a muito baixa estatura mais frequentes entre os meninos em ambas as faixas etárias. Com relação à classificação do peso-para-idade, notou-se que o peso elevado apresentou maior prevalência em meninas, principalmente nas crianças menores de cinco anos (17,8%). Para as crianças de 5-9 anos, as prevalências entre os sexos ficaram mais próximas (23,4% em meninas vs. 22,5% em meninos) (Figura 2).

Na Figura 3, que avalia o IMC-para-idade, o risco de sobrepeso, o sobrepeso e a obesidade foram mais prevalentes em meninas menores de cinco anos, com destaque para o risco de sobrepeso (26,6%). Entre as crianças de 5-9 anos, o risco de sobrepeso, o sobrepeso e a obesidade apresentaram valores mais altos entre os meninos, especialmente para o sobrepeso (18,7%) e a obesidade grave (10,5%).

Com relação à dupla carga de má nutrição, a prevalência total encontrada na amostra foi de 4,7%. Estratificando-se por faixa etária, observou-se frequência maior da dupla carga de má nutrição em crianças menores de cinco anos (6,5%). Entre os menores de cinco anos, a dupla carga de má nutrição foi mais prevalente nos meninos em ambas as faixas etárias. Com relação aos parâmetros utilizados na classificação da dupla carga de má nutrição, o excesso de peso foi levemente mais frequente em crianças de 5-9 anos, com prevalências semelhantes entre os sexos. A baixa estatura foi mais prevalente nos menores de cinco anos, destacando-se a maior frequência nos meninos (17,5%) (Figura 4).

**Tabela 1:** Classificação do estado nutricional das crianças atendidas nas unidades básicas de saúde de acordo com a faixa etária. Icapuí, Ceará, 2023 (n=1704)

Variáveis	Total	Crianças <5 anos	Crianças de 5-9 anos	n (%)	n (%)	n (%)
Peso-para-estatura^a^			
Magreza acentuada	27 (3,3)	27 (3,3)	-
Magreza	29 (3,6)	29 (3,6)	-
Eutrofia	374 (46,2)	374 (46,2)	-
Risco de sobrepeso	184 (22,8)	184 (22,8)	-
Sobrepeso	102 (12,6)	102 (12,6)	-
Obesidade	91 (11,3)	91 (11,3)	-
Estatura-para-idade			
Muito baixa	94 (5,5)	49 (6)	45 (5)
Baixa	80 (4,7)	50 (6,2)	30 (3,4)
Adequada	1530 (89,8)	711 (87,8)	819 (91,6)
Peso-para-idade			
Muito baixo	40 (2,3)	11 (1,4)	29 (3,2)
Baixo	35 (2,1)	20 (2,5)	15 (1,7)
Adequado	1289 (76,2)	653 (80,6)	645 (72,1)
Elevado	331 (19,4)	126 (15,6)	205 (22,9)
IMC-para-idade^b^			
Magreza acentuada	48 (2,8)	33 (4,1)	15 (1,7)
Magreza	55 (3,2)	24 (3)	31 (3,5)
Eutrofia	814 (47,8)	370 (45,7)	444 (49,7)
Risco de sobrepeso	211 (12,4)	180 (22,2)	31 (3,5)
Sobrepeso	264 (15,5)	106 (13,1)	158 (17,7)
Obesidade	231 (13,6)	97 (12)	134 (15)
Obesidade grave^c^	81 (4,8)	-	81 (9,1)

a Parâmetro disponível apenas crianças menores de 5 anos (n=807); ^b^Índice de massa corporal-para-idade; ^c^Classificação disponível apenas para crianças a partir de 5 anos.

**Figura 1:** Classificação do peso-para-estatura das crianças menores de cinco anos (parâmetro disponível apenas para essa faixa etária) atendidas nas unidades básicas de saúde, de acordo com o sexo. Icapuí, Ceará, 2023 (n=807)

**Figura 2:** Classificação da estatura-para-idade das crianças menores de cinco anos (A) e de 5-9 anos (B); classificação do peso-para-idade das crianças menores de cinco anos (C) e de 5-9 anos (D) atendidas nas unidades básicas de saúde, de acordo com o sexo. Icapuí, Ceará, 2023 (n=1704)

**Figura 3:** Classificação do índice de massa corporal-por-idade (IMC-por-idade) das crianças menores de cinco anos (A) e de 5-9 anos (B) atendidas nas unidades básicas de saúde, de acordo com o sexo. Icapuí, Ceará, 2023 (n=1704)

**Figura 4:** Prevalência de Dupla Carga de Má Nutrição (DCMN) e dos critérios de classificação da DCMN para crianças menores de cinco anos (A) e de 5-9 anos (B) atendidas nas unidades básicas de saúde, de acordo com o sexo. Icapuí, Ceará, 2023 (n=1704)

## Discussão 

Este estudo revelou uma prevalência importante da dupla carga de má nutrição em crianças, evidenciando a coexistência de condições nutricionais opostas, como o excesso de peso e a baixa estatura. Este levantamento constitui um estudo pioneiro no Ceará, com uma amostra relevante do interior do estado, destacando-se por explorar a complexa coexistência de condições de má nutrição na infância e a sua relação com o contexto e as tendências atuais ^10^.

Sabe-se das limitações inerentes aos estudos com dados secundários, relacionadas a inconsistências e a incompletudes em alguns dados coletados, além da ausência de integração direta entre as informações sobre filho e mãe (incluindo-se seus estados nutricional e socioeconômico), o que impediu a realização de associações sobre os determinantes da dupla carga de má nutrição ^11^. Mesmo assim, é importante considerar que a escolha metodológica de utilizar exclusivamente o IMC-para-idade como indicador de peso para todas as faixas etárias, embora alinhada às recomendações do SISVAN e coerente com estudos semelhantes, pode ter gerado diferenças pontuais em relação a outros indicadores (como peso-para-idade para menores de dois anos). Essa decisão visou garantir comparabilidade entre faixas etárias e permitir a avaliação simultânea de baixa estatura e de excesso de peso em toda a amostra. Além disso, o SISVAN, por abranger apenas crianças da rede pública de saúde, pode afetar a generalização dos resultados, embora sua cobertura nacional venha se expandindo ^12^. Quanto à qualidade dos dados, os protocolos padronizados da Norma Técnica, de 2011, ^13^ asseguram maior confiabilidade às medidas antropométricas, mesmo com possíveis variações na coleta.

Diante dessa realidade, ainda que estudos específicos sobre a prevalência global de dupla carga de má nutrição sejam limitados, dados regionais fornecem percepções importantes. Na África, um estudo, realizado em dois estados nigerianos, com 1.200 crianças e adolescentes, foi encontrada uma prevalência de nanismo de 34,9% e de sobrepeso/obesidade de 11,4%. Assim, apresentaram 4,0% de dupla carga de má nutrição em nível individual, variando conforme as características demográficas, socioeconômicas e familiares ^14^. No continente sulamericano, em pesquisas recentes feitas com crianças e adultos, a prevalência de dupla carga de má nutrição em domicílios na Bolívia foi de 9,3%, enquanto no Peru foi registrada uma prevalência de 6,7%. Na Colômbia, por sua vez, a prevalência foi de 3,2%, já no Brasil foi de 2,2% ^15^.

No Brasil, condições maternas, como o estado nutricional e o nível de escolaridade, influenciam o estado nutricional das crianças, contribuindo para a ocorrência da dupla carga de má nutrição, como evidenciado em estudo de crianças menores de cinco anos em diferentes regiões do país, incluindo zonas urbanas e rurais ^16^.

No Nordeste brasileiro, estudos contendo informações de 50.778 crianças e adolescentes (0-14 anos) apontaram uma média de desnutrição, de sobrepeso e de obesidade de 1,9%, 12,5% e 7,4%, respectivamente. Conforme os trabalhos analisados, os resultados indicaram também fatores socioeconômicos, como moradia e renda, ligados ao estado nutricional ^17^.

Em um estudo transversal realizado em dois municípios da Paraíba, das 413 crianças que participaram do estudo, 9,4% foram diagnosticadas com déficit de estatura e 16,5% com sobrepeso/obesidade. Dessa forma, a baixa estatura infantil pode ser associada ao peso ao nascer, à idade gestacional, ao estado nutricional materno e ao nível socioeconômico da família. Já quanto ao excesso de peso observou-se relação com a idade da criança, o IMC materno, o benefício do Bolsa Família e o suporte social ^5^^,^^18^.

O presente estudo revelou que a maioria das crianças icapuienses apresentava peso adequado, mas uma proporção importante apresentava sobrepeso ou obesidade, especialmente nas faixas etárias mais avançadas. Além disso, a baixa estatura, que é um indicativo de desnutrição crônica, particularmente entre as crianças menores de cinco anos, sugere a necessidade de intervenções para melhorar as condições de saúde e garantir o crescimento adequado desde a primeira infância ^19^^,^^20^. Em termos de diferenças entre as faixas etárias, a prevalência de dupla carga de má nutrição foi maior entre as crianças menores de cinco anos em comparação às crianças de 5-9 anos. A influência do sexo é evidente, com o sexo masculino apresentando maior prevalência de dupla carga de má nutrição, de excesso de peso e de baixa estatura em ambas as faixas etárias. Essa disparidade pode estar relacionada a diferenças biológicas ou culturais que afetam a saúde nutricional entre meninos e meninas, sendo necessário explorar práticas alimentares, acesso a alimentos e cuidados gerais em estudos adicionais ^21^^-^^23^.

Entre as crianças mais velhas, o excesso de peso foi mais prevalente, sugerindo que o ganho de peso desproporcional ocorre em idades mais avançadas. Em crianças menores de cinco anos, notou-se uma maior prevalência de magreza e de baixa estatura, o que pode ser atribuído a fatores socioeconômicos e a acesso limitado a serviços de saúde ^10^.

A desnutrição e o excesso de peso, ambos componentes da dupla carga de má nutrição, estão frequentemente associados a condições de insegurança alimentar, o que leva as famílias a consumirem alimentos de baixo valor nutricional e alta densidade calórica, como ultraprocessados. Isso resulta em deficiências nutricionais, com possível comprometimento de crescimento, além de ganho de peso excessivo. Esse quadro reflete a transição nutricional e epidemiológica, marcada por mudanças nos padrões alimentares e pelo sedentarismo ^24^^-^^27^.

Quanto ao comprometimento do crescimento linear, os resultados apontam para uma maior vulnerabilidade no sexo masculino em ambas as faixas etárias. Essa situação pode ser explicada por fatores biológicos, como maior suscetibilidade dos meninos a condições adversas nos primeiros anos de vida, ou por fatores ambientais, como acesso desigual a alimentos nutritivos e a serviços de saúde ^22^.

Entre meninas, os expressivos percentuais de excesso de peso podem estar associados a fatores biológicos, como predisposição ao acúmulo de gordura, além de aspectos culturais e sociais, como menor incentivo à prática de atividades físicas. Comportamentos alimentares relacionados à alimentação emocional também contribuem, assim como a falta de políticas preventivas inclusivas que considerem as diferenças de gênero ^25^.

A persistência de déficits de crescimento em um grupo considerável de crianças, especialmente nas áreas de maior vulnerabilidade social, destaca a importância de políticas públicas voltadas para a segurança alimentar e a nutricional, focando-se, principalmente, sobre o período inicial da infância e as regiões com difícil acesso a alimentos nutritivos ^28^. Esse cenário exige intervenções que promovam tanto uma alimentação equilibrada quanto a prática de atividade física regular. Especialmente nas escolas, que desempenham um papel importante na promoção de hábitos alimentares saudáveis e no controle do consumo de alimentos hipercalóricos. Esses fatores socioeconômicos e ambientais reforçam a necessidade de uma abordagem de forma integrada para prevenir tanto o excesso de peso quanto a desnutrição ^21^^,^^13^.

Este estudo se destaca por sua abrangência, contemplando cerca de 88% das crianças do município investigado, um alcance significativo quando comparado ao total de crianças de Icapuí ^7^. Além disso, representa um marco importante na investigação da dupla carga de má nutrição no Nordeste brasileiro, particularmente no interior do estado do Ceará, preenchendo uma lacuna importante na literatura regional.

Os achados deste estudo permitem identificar a necessidade de intervenções integradas e contínuas que abordem tanto a prevenção quanto o tratamento de problemas nutricionais com foco em populações vulneráveis. Políticas públicas devem priorizar a promoção da segurança alimentar e nutricional, estimular práticas alimentares saudáveis desde a infância e ampliar a cobertura de programas como o SISVAN, integrando estratégias voltadas à educação nutricional, ao acesso a alimentos saudáveis e ao incentivo à atividade física. Além disso, as escolas desempenham um papel fundamental nesse contexto, podendo servir como agentes de mudança para a adoção de hábitos mais saudáveis entre as crianças ^16^^,^^17^.

Pode-se concluir que a dupla carga de má nutrição é uma realidade epidemiológica em crianças atendidas pela rede de saúde pública de Icapuí, Ceará, com padrões distintos: menores de cinco anos são mais vulneráveis ao déficit estatural, enquanto crianças escolares (5-9 anos) o são ao excesso de peso; além de haver disparidades de gênero, tendo os meninos maior risco de baixa estatura e as meninas de sobrepeso. Esses achados fornecem subsídios para o planejamento de intervenções mais efetivas, destacando a necessidade de respostas articuladas que enfrentem, simultaneamente, os desafios tradicionais e os emergentes da nutrição infantil no Nordeste brasileiro. A complexidade da dupla carga exige abordagens inovadoras que transcendam as soluções convencionais, integrando saberes locais às evidências científicas, a fim de desenvolver estratégias que possam reverter o cenário. 

## Data Availability

Os dados que corroboram os resultados deste estudo estão disponíveis mediante solicitação ao autor correspondente.
